# Vagally Mediated Heart Rate Variability and Attachment-Related Avoidance in Pediatric Migraine: A Mediated Pathway to Somatic Symptoms Severity

**DOI:** 10.3390/children12121602

**Published:** 2025-11-25

**Authors:** Filippo Cellucci, Chiara Morale, Giulia Di Vincenzo, Giovanni Di Nardo, Alessandro Ferretti, Pasquale Parisi, Valeria Carola, Giampaolo Nicolais

**Affiliations:** 1 Department of Dynamic, Clinical and Health Psychology, Faculty of Medicine and Psychology, Sapienza University of Rome, 00185 Rome, Italyvaleria.carola@uniroma1.it (V.C.); giampaolo.nicolais@uniroma1.it (G.N.); 2 Neuroscience, Mental Health and Sense Organs (NESMOS) Department, Faculty of Medicine and Psychology, Sapienza University of Rome, 00189 Rome, Italyalessandro.ferretti@uniroma1.it (A.F.); pasquale.parisi@uniroma1.it (P.P.)

**Keywords:** pediatric migraine, somatic symptoms, attachment avoidance, vagally mediated heart rate variability, parent–child relationship

## Abstract

**Highlights:**

**What are the main findings?**

- Attachment-related anxiety and avoidance toward caregivers are associated with severity of somatic symptoms in pediatric migraine.
- Avoidant attachment toward the father fully mediates the relationship between resting vagally mediated heart rate variability and somatic symptom severity in children and adolescents with migraine.

**What are the implications of the main findings?**

- Relational factors, especially attachment dynamics, play a central role in understanding somatic distress in pediatric migraine.
- Targeting both family relationships and physiological regulation may offer added value to current non-pharmacological treatments for pediatric migraine.

**Abstract:**

**Background/Objectives**: Pediatric migraine is a prevalent and disabling condition often accompanied by functional somatic symptoms and emotional dysregulation. Emerging evidence suggests that autonomic imbalance and insecure attachment patterns may both contribute to the development and maintenance of somatic distress. However, the interplay between physiological regulation and relational dynamics remains insufficiently understood, particularly in pediatric clinical populations. This study investigated whether attachment-related anxiety and avoidance toward both mother and father and resting Vagally Mediated Heart Rate Variability (vmHRV) were associated with somatic symptom severity in adolescents with migraine. Additionally, it tested whether attachment dimensions mediate the association between resting vmHRV and somatic symptoms. **Methods**: Sixty-one adolescents (aged 11–17 years) with a clinical diagnosis of migraine completed self-report measures assessing somatic symptoms (CSI-24) and attachment dimensions toward each parent (ECR-RC). Resting vmHRV (RMSSD) was recorded during a five-minute baseline. Correlational analyses, multiple regressions, and bootstrapped mediation models were conducted. **Results**: Higher somatic symptom severity was significantly associated with both attachment anxiety and avoidance toward both parents. Regression models showed that attachment anxiety and avoidance to the mother, along with attachment avoidance to the father, predicted somatic symptoms. Although vmHRV was not directly associated with symptom severity, mediation analysis revealed that attachment avoidance to the father fully mediated the relationship between lower resting vmHRV and increased somatic complaints. **Conclusions**: These findings highlight the relevance of relational factors in pediatric migraine and suggest that avoidant attachment—particularly toward the father—may serve as a psychological mechanism linking autonomic dysregulation to somatic symptomatology. The results support integrative, biopsychosocial models for understanding and treating primary headache in youth, emphasizing the potential of combining attachment-focused and physiological interventions in clinical practice.

## 1. Introduction

Primary headache disorders—including migraine, tension-type headache, trigeminal autonomic cephalalgias, and other primary headache disorders—account for the vast majority of headache-related consultations in specialized clinical settings [1]. In the pediatric population, although epidemiological data are not entirely consistent, the prevalence of primary headaches has increased significantly during childhood and adolescence in recent years. Some authors have reported a prevalence of primary headaches of approximately 54.4% in pediatric patients [2]. It is conceptualized as a significant psychosocial burden, influencing academic achievement, peer relationships, and emotional development [3]. While headaches are relatively uncommon before age four, their prevalence rises markedly throughout development: from about 5% in preschool years to 37% in early school age, reaching up to 75% during adolescence [4,5]. Gender differences have also been consistently observed, with a female-to-male ratio of 1.5:1 before age 10, increasing to 3:1 in adolescence, indicating a disproportionately higher prevalence in females during later developmental stages [4].

From a neurobiological perspective, migraine is traditionally conceptualized as a neurovascular disorder with a strong genetic component [6]. Evidence in pediatric populations remains limited; however, a twin family study documented heritable influences on pediatric primary pain disorders, with migraine showing the strongest genetic contribution among the conditions examined [7]. Neuroimaging studies have identified altered activity in pain-related regions such as the anterior cingulate cortex, prefrontal cortex, and supplementary motor area, indicating impaired top-down pain modulation [8,9,10,11]. Genetic research further supports this view, with recent large-scale genome-wide association studies identifying more than 178 genomic loci associated with migraine, thereby underscoring its complex polygenic architecture [12]. Beyond genetic predispositions, psychosocial influences are increasingly acknowledged as central to understanding both the onset and the persistence of pediatric migraine. Accumulating evidence indicates that pediatric migraine is most appropriately conceptualized within a biopsychosocial framework, emphasizing the dynamic interaction among neurobiological, psychological, and environmental factors. Children with migraine are more likely to experience emotional dysregulation, anxiety, somatization, and depressive symptoms [8,13]. Psychosocial stressors—including parental psychopathology, stressful life events, and a history of adverse childhood experiences (ACEs)—have been linked to an increased risk of both onset and chronicity of migraine [8,14,15,16,17,18,19]. For example, maternal depression, poor familial support, and excessive parental control have all been associated with greater migraine severity and frequency in youth [13,20], highlighting the need to account for the relational context in which symptoms arise and are managed.

Attachment Theory offers a compelling framework for understanding how early caregiving experiences shape a child’s stress, emotion, and pain regulation. Children with insecure attachment styles—particularly avoidant or anxious-ambivalent—may have greater difficulty recognizing and articulating emotional distress, which can be somatically expressed through physical symptoms, including migraine and other forms of functional pain [20,21]. Avoidant attachment, in particular, has been associated with a tendency to suppress affect and communicate discomfort through bodily channels [22,23]. In contrast, ambivalent attachment may intensify the affective response to pain due to heightened sensitivity to negative emotions [24,25]. In pediatric migraine populations, high rates of insecure attachment have been reported [26,27], further reinforcing the need to explore relational dynamics in clinical assessments. Despite these findings, a notable gap in the literature concerns the differential role of attachment to mothers versus attachment to fathers. Most studies focus predominantly on attachment relationships with mothers, overlooking the unique and non-interchangeable contribution of paternal bonding to child development [28]. However, recent studies suggest that avoidant attachment to fathers may play a distinct role in shaping emotional and somatic outcomes in children with migraine [29,30]. Understanding this dimension is essential for refining developmental pain models and tailoring interventions that engage the broader family system.

From a psychophysiological standpoint, the autonomic nervous system (ANS) activity—particularly vagal tone —has emerged as a promising biomarker in the study of chronic pain conditions, including migraine [31,32]. The ANS regulates physiological responses to stress, and an imbalance between sympathetic and parasympathetic branches is frequently observed in migraine patients [32]. However, most of the available evidence pertains to adult populations. Among the different types of ANS activity measurement, Heart Rate Variability (HRV) indicates the variation in the time interval between heartbeats. This is measured by the variation in the interval between two successive beats and could also be considered as a non-invasive index of the organism’s capacity for the adaptive regulation of emotional and physiological states [33,34]. Lower resting HRV has been linked to increased vulnerability to stress and pain as well as to emotional dysregulation and disorganized attachment patterns [35,36].

Among the different HRV indices, vagally mediated heart rate variability (vmHRV) refers to the portion of short-term heart rate variability that is primarily driven by tonic and phasic parasympathetic (vagal) influences on the sino-atrial node. vmHRV is widely used as a non-invasive index of cardiac vagal control and the organism’s capacity for flexible physiological and emotional regulation. Recent syntheses emphasize vmHRV as an actionable biomarker for self-regulatory capacity and as the most appropriate HRV construct when the research focuses on parasympathetic functioning [37]. Its application in pediatric and adolescent samples has been well-established, with developmental studies demonstrating normative changes in parasympathetic function and clinical research supporting its relevance for emotional health [38,39]. Previous research has outlined five major frameworks linking HRV to psychophysiological processes [37,38]: the neurovisceral integration model [33], the polyvagal theory [35], the biological behavioral model [39], the resonance frequency model [40], and the psychophysiological coherence model [41]. Despite their differences, these perspectives converge on the central role of vagal tone, which remains a key focus of HRV research [37]. For instance, in the context of migraine, several studies have reported evidence of vagal imbalance, often reflected in reduced vmHRV [42,43,44]. Although research directly addressing vagal activity in pediatric populations remains limited, autogenic training and biofeedback—commonly recommended in managing pediatric headaches—are specifically designed to target autonomic regulation, aiming to restore vagal balance and enhance emotional self-regulation [45,46]. Taken together, the available evidence points to the complex interplay between neurobiological vulnerability, emotional functioning, attachment relationships, and autonomic regulation in pediatric migraine. However, the integration of these components into a unified explanatory model remains limited. To our knowledge, few studies have explored how attachment to both parents, autonomic functioning, and somatic symptom severity interrelate in children and adolescents with migraine.

In sum, pediatric migraine represents a paradigmatic condition for examining the dynamic interplay between biological vulnerability, emotional–relational factors, and ANS activity. Although the interconnections among these domains are increasingly acknowledged, there is still a paucity of integrative frameworks that jointly address attachment relationships, vagal modulation, and somatic symptomatology in pediatric populations. Notably, the specific roles of attachment to mothers and fathers in mediating or moderating the association between vagal tone and somatic symptom severity have received limited attention. Addressing this gap is crucial for advancing a more refined and developmentally sensitive understanding of individual differences in pediatric pain expression and informing prevention and intervention strategies that explicitly consider the child’s relational context.

The present study examines in pediatric population with migraine:

(a) The *associations* between somatic symptoms, attachment patterns to mother and father, and resting vmHRV.
(b) If attachment patterns to mother and father, resting HRV and gender *predict* independently somatic symptoms.
(c) If attachment patterns to parents *mediate* the relationship between HRV and somatic symptoms.

## 2. Materials and Methods

### 2.1. Participants and Procedure

The present study was conducted in accordance with the ethical principles outlined in the Declaration of Helsinki for research involving human participants. Ethical approval was obtained from the CET Lazio Area Ethics Committee (Rif. 797—6 August 2025). All participants were assessed as part of standard clinical psychological evaluations performed in a hospital setting for pediatric migraine management. Parents or legal guardians provided written informed consent for the assessment and for the anonymous use of clinical data for research purposes, and assent was obtained from each child. Both parents and minors were informed of their right to withdraw from the procedure and study at any time without any adverse consequence. Families were informed, both verbally and in writing, that refusal to authorize the anonymous use of data for research purposes would not affect the clinical psychological assessment or the care received. No financial compensation or material incentives were provided to participating families.

Participants who met the eligibility criteria and whose parents provided written informed consent for the clinical assessment and the anonymous use of clinical data for research purposes were recruited from the Pediatric Unit (UOC di Pediatria) of Sant’Andrea University Hospital in Rome. Inclusion criteria were as follows:

(a) a diagnosis of migraine based on the International Classification of Headache Disorders, 3rd edition (ICHD-III);
(b) age between 11 and 17 years;
(c) adequate comprehension of the Italian language;
(d) absence of comorbid neurological conditions.

Initial screening was conducted by medical personnel who identified eligible patients according to ICHD-III diagnostic criteria. As part of the routine day-hospital multidisciplinary assessment, a licensed clinical psychologist met with each patient to perform the clinical psychological evaluation (this assessment was conducted first during the day-hospital visit).

After obtaining parental consent and child assent, physiological recordings were obtained at the beginning of the session, during a 5 min resting baseline with the participant seated quietly. This was done prior to the clinical interview and questionnaire administration to ensure that emotional or cognitive engagement with the assessment content did not influence autonomic parameters. Subsequently, the clinical interview was conducted, followed by completion of the self-report questionnaires. All procedures took place during the same day-hospital visit.

### 2.2. Psychometric Measures

#### 2.2.1. The Children’s Somatization Inventory-24 (CSI-24), Italian Version

Somatic symptomatology was assessed using the Italian version of the Children’s Somatization Inventory—24 [47,48], a self-report measure designed to evaluate the presence and severity of physical symptoms in children and adolescents over the previous two weeks. The instrument consists of 24 items covering a range of somatic complaints, including headaches, abdominal pain, dizziness, nausea, and musculoskeletal discomfort. Items are rated on a 5-point Likert scale (0 = “Not at all” to 4 = “A whole lot”), yielding a total score ranging from 0 to 96. Higher scores indicate greater frequency and severity of symptoms. Empirical research supports a bifactorial structure, distinguishing between Gastrointestinal (GI) and Non-Gastrointestinal (non-GI) subscales [47]. The Italian validation study reported excellent internal consistency (α = 0.91) [29] and satisfactory convergent validity through correlations with alexithymia and depressive [47,49]. The CSI-24 is considered a valid and reliable tool for assessing somatic symptoms in pediatric populations, with demonstrated utility in clinical and community settings [48].

#### 2.2.2. Experiences in Close Relationships—Revised Child Version (ECR-RC-12), Italian Version

Attachment-related anxiety and avoidance to mothers and fathers were assessed using the Italian short form of the Experiences in Close Relationships Scale—Revised Child version—ECR-RC-12—[50,51]. Derived from a widely used adult measure, the ECR-RC-12 consists of 12 self-report items adapted for use with children and early adolescents. The instrument includes two subscales—Anxiety and Avoidance—each comprising six items written in developmentally appropriate language. The Anxiety subscale assesses fears regarding the availability and responsiveness of attachment figures, while the Avoidance subscale captures discomfort with closeness and emotional dependence. Responses are given on a 5-point Likert scale ranging from 1 (“Not at all like me”) to 5 (“Very much like me”). Two parallel forms of the questionnaire assess attachment representations toward the mother and father separately, allowing for a differentiated evaluation of attachment relationships. The Italian validation study, conducted with 448 children aged 8 to 13, confirmed a robust two-factor structure, strong measurement invariance across age groups, and excellent internal consistency for both parental versions (α > 0.80) [51]. Participants were instructed to complete the ECR-RC measures with reference to their biological father, regardless of the type or quality of the paternal relationship. As noted in the sample size reported for the analysis model, four children were excluded from the analyses because they did not respond to the item regarding their biological father, due either to high levels of distancing or lack of recognition.

### 2.3. Psychophysiological Measurement

#### Resting Vagally Mediated Heart Rate Variability (vmHRV)

Vagally mediated HRV (vmHRV) was assessed using the root mean square of successive differences (RMSSD, ms), computed from beat-to-beat intervals recorded with the Bodyguard 2 device (Firstbeat Technologies, Finland). This device strongly agrees with standard 5-lead electrocardiograms (ECG) regarding HRV measurement accuracy [52]. RMSSD was selected as the primary index because it is a validated time-domain measure of parasympathetic (vagal) cardiac influence, suitable for short-term baseline recordings, and less sensitive to respiratory confounds than specific frequency-domain indices [37,53]. Continuous R–R interval data were collected during a 5 min seated baseline epoch, with all recordings conducted between 9:00 a.m. and 11:30 a.m. Participants were instructed to refrain from heavy meals and from consuming caffeine- or theine-containing beverages for at least two hours before measurement to minimize confounding influences. Although vmHRV was continuously monitored across the entire session, resting vmHRV was operationalized as the RMSSD computed over the five-minute baseline period, during which participants remained seated and received no task instructions. Data processing included standardized artifact correction (“very low” setting) as recommended in recent methodological guidelines [37,54]. vmHRV analyses were performed using Kubios HRV software [54].

### 2.4. Statistical Analysis

An a priori power analysis was conducted using G*Power version 3.1.9.7 [55] to determine the minimum required sample size for a multiple linear regression (fixed model, R^2^ deviation from zero). Assuming a medium-to-large effect size (f^2^ = 0.30), α = 0.05, power (1–β) = 0.90, and three predictors, the required sample size was 52 (actual power = 0.904), with 48 degrees of freedom in the denominator. The noncentrality parameter (λ) was 15.60, and the critical F-value was 2.798.

First, Pearson’s correlations were computed to examine associations among somatic symptom severity (CSI-24), attachment-related anxiety and avoidance toward the mother and father (ECR-RC), and resting vmHRV (RMSSD).

Based on significant associations with CSI-24, three multiple linear regression models were conducted to assess the independent predictive value of:

- Attachment to mother dimensions (anxiety and avoidance, ECR-RC) and gender;
- Attachment to father dimensions (anxiety and avoidance, ECR-RC) and gender;
- Resting vmHRV (RMSSD), body mass index (BMI), and gender.

All models were estimated using the enter method. Unstandardized coefficients (B), standard errors (SE), 95% confidence intervals (CIs), and *p*-values were reported for each predictor. Model fit was evaluated using R, R^2^, and adjusted R^2^. The assumptions of linearity, normality of residuals, and homoscedasticity were checked and met for all models.

Finally, a mediation analysis was conducted within a path-analytic framework to investigate potential mechanisms linking autonomic regulation and somatic symptomatology (https://jamovi-amm.github.io/, accessed on 28 July 2025). This model tested whether attachment avoidance and anxiety to mother and father mediated the association between resting vmHRV (RMSSD) and somatic symptoms (CSI-24). All variables were entered as observed measures, and completely standardized coefficients (β) were reported. The significance of the indirect effect was evaluated using a bias-corrected bootstrapping procedure with 5000 resamples, and 95% CIs were examined to determine whether they excluded zero. Direct and total effects were also calculated to assess the presence of full or partial mediation.

In summary, the statistical workflow moved from descriptive and correlational analyses to targeted multiple regression models and finally to a mediation model.

## 3. Results

At the end of recruitment, a total of 63 patients met the study eligibility criteria. Two participants were excluded because parental consent for the use of clinical data for research purposes was not provided. No exclusions occurred due to language comprehension difficulties or neurological comorbidities. The final sample comprised 61 participants (37 females and 24 males), with a mean age of 13.9 years (SD = 1.98; range: 11–17). The mean age was 14.7 years for females (SD = 1.61; range: 12–17) and 12.6 years for males (SD = 1.86; range: 11–17). The mean body mass index (BMI) was 23.7 (SD = 5.35) for the total sample, with females reporting a mean BMI of 23.6 (SD = 5.23) and males 23.8 (SD = 5.65). Additional demographic characteristics are presented in Table 1.

### 3.1. Associations Between Somatic Symptoms, Attachment Patterns to Mother and Father, and Resting vmHRV

Pearson’s correlations were conducted to explore associations among somatic symptoms, attachment patterns to mother and father, and resting vmHRV (see Table 2).

The results showed that somatic symptoms were positively correlated with ECR-RC Anxiety Mother (r = 0.422, *p* < 0.001), ECR-RC Avoidance Mother (r = 0.465, *p* < 0.001), ECR-RC Anxiety Father (r = 0.334, *p* < 0.05), and ECR-RC Avoidance Father (r = 0.444, *p* < 0.001).

Moreover, while RMSSD—Baseline was not directly associated with somatic symptoms (r = 0.034, ns), ECR-RC Avoidance Father was negatively correlated with RMSSD—Baseline (r = −0.330, *p* < 0.05). No other attachment dimensions (ECR-RC Anxiety or Avoidance with mothers and ECR-RC Anxiety with fathers) were significantly correlated with RMSSD—Baseline.

### 3.2. Assessment of Attachment Patterns to Mother and Father, Resting vmHRV, and Gender as Predictors of Somatic Symptoms

#### 3.2.1. Attachment to Mother and Gender as Predictors of CSI-24

The linear regression model, estimated with a sample size of 61 participants, explained 28.8% of the variance in CSI-24 scores (R = 0.536, R^2^ = 0.288). Results (see Table 3) indicated that higher scores on ECR-RC Anxiety Mother (B = 1.066, SE = 0.506, t = 2.10, *p* = 0.040) and ECR-RC Avoidance Mother (B = 0.694, SE = 0.290, t = 2.39, *p* = 0.020) were significantly associated with higher CSI-24 scores. Gender (male vs. female) was not a significant predictor (B = −3.530, SE = 3.202, t = −1.10, *p* = 0.275). The model intercept was 6.787 (SE = 5.291, t = 1.28, *p* = 0.205).

#### 3.2.2. Attachment to Father and Gender as Predictor of CSI-24

The linear regression model, estimated with a sample size of 57 participants, accounted for 24.9% of the variance in CSI-24 scores (R = 0.499, R^2^ = 0.249). ECR-RC Avoidance Father was a significant predictor (B = 0.669, SE = 0.327, t = 2.05, *p* = 0.046), whereas ECR-RC Anxiety Father (B = 0.863, SE = 0.515, t = 1.68, *p* = 0.099) and gender (male vs. female; B = −4.574, SE = 3.586, t = −1.28, *p* = 0.208) were not statistically significant predictors (see Table 4). The model intercept was 7.965 (SE = 6.016, t = 1.32, *p* = 0.191).

#### 3.2.3. vmHRV, BMI, and Gender as Predictors of CSI-24

The linear regression model explained 8.25% of the variance in CSI-24 scores (R = 0.287, R^2^ = 0.0825). Neither RMSSD—Baseline (B = −0.0408, SE = 0.0934, t = −0.437, *p* = 0.664) nor BMI (B = 0.1626, SE = 0.3290, t = 0.494, *p* = 0.623) were significant predictors (see Table 5). The model intercept was 25.0559 (SE = 9.5964, t = 2.611, *p* = 0.012). Among the predictors, gender (male vs. female) was statistically significant (B = −7.2141, SE = 3.5815, t = −2.014, *p* = 0.049).

### 3.3. Mediation Model: Attachment Patterns to Mothers and Fathers as Potential Mediators of the Effect of Resting vmHRV on Somatic Symptoms

A series of mediation analyses was conducted using a path analytic framework to test the third hypothesis (c) that attachment patterns mediate the association between resting vmHRV and somatic symptomatology. In line with the regression findings, the attachment dimensions that significantly predicted somatic symptoms (i.e., ECR-RC Anxiety Mother, ECR-RC Avoidance Mother, ECR-RC Avoidance Father) were initially considered as potential mediators of the association between baseline RMSSD—Baseline and CSI-24 scores. Among these models, only the one that included avoidance to father as a mediator reached statistical significance.

**Figure 1:**
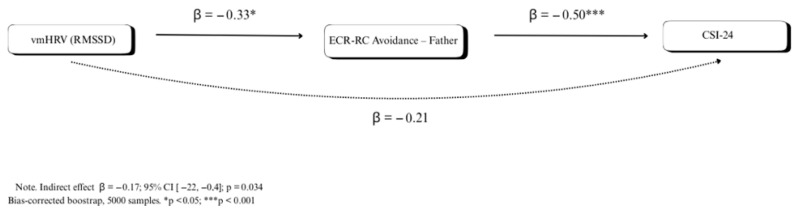
Attachment avoidance to fathers as a mediator of the effect of resting vmHRV on CSI-24.

The mediation analysis (see Table 6) tested whether attachment avoidance to fathers (ECR-RC Avoidance Father) mediated the association between baseline RMSSD and somatic symptom severity (CSI-24 total score). Confidence intervals were computed using a bias-corrected bootstrap, and all coefficients are reported as completely standardized effect sizes. The indirect effect was statistically significant (Estimate = −0.1050, SE = 0.0496, 95% CI [−0.2159, −0.0355], β = −0.1661, z = −2.116, *p* = 0.034). The first path of the indirect effect indicated that baseline RMSSD was negatively associated with attachment avoidance to fathers (Estimate = −0.0946, SE = 0.0376, 95% CI [−0.1568, −0.0367], β = −0.3298, z = −2.519, *p* = 0.012). The second path showed that attachment avoidance to fathers was positively associated with CSI-24 scores (Estimate = 1.1100, SE = 0.2844, 95% CI [0.5955, 1.6710], β = 0.5037, z = 3.902, *p* < 0.001). The direct effect of baseline RMSSD—Baseline on CSI-24 was not statistically significant (Estimate = 0.1302, SE = 0.0816, 95% CI [−0.0717, 0.2997], β = 0.2060, z = 1.596, *p* = 0.111). The total effect was also non-significant (Estimate = 0.0217, SE = 0.0851, 95% CI [−0.1784, 0.1835], β = 0.0343, z = 0.254, *p* = 0.799). A graphical simplification can be seen in Figure 1.

## 4. Discussion

The present study investigated, in a pediatric population with migraine, (a) the associations between somatic symptoms (CSI-24), attachment patterns to mother and father (ECR-RC), and resting vmHRV (RMSSD); (b) the independent predictive value of attachment dimensions, resting vmHRV, and gender on somatic symptoms; and (c) the potential mediating role of attachment patterns to mothers and fathers in the relationship between resting vmHRV and somatic symptoms.

Consistent with our first hypothesis, we observed significant positive correlations between somatic symptom severity and attachment-related anxiety and avoidance toward both parents, with effect sizes in the moderate range. Notably, both anxiety and avoidance to mothers and fathers were associated with higher somatization scores. These findings align with prior evidence linking insecure attachment patterns to the experience and reporting of somatic complaints in pediatric and adolescent populations [20,22,56]. Importantly, resting vmHRV (indexed by RMSSD) was not directly associated with somatic symptoms, but it showed a significant negative correlation with attachment avoidance to fathers. This pattern suggests that reduced vagal tone may be particularly relevant in the context of relational schemas involving emotional disengagement from the father figure. From the framework of biopsychosocial models of pain, which posit that physiological markers of stress vulnerability—such as low HRV—may impact symptom expression when embedded in maladaptive relational or emotional contexts [8,35], these findings underscore the need to consider both autonomic regulation and the quality of parent–child relationships as intertwined factors that may shape the manifestation of somatic symptoms in pediatric migraine. Previous research has identified such autonomic patterns as correlates of avoidant attachment, reflecting a diminished capacity for physiological co-regulation [23,25,57]. Regression models further clarified these associations. When attachment to the mother was considered, both anxiety and avoidance predicted higher somatic symptoms, whereas gender was not significant. In contrast, in the paternal model, only avoidance emerged as a significant predictor, with anxiety showing a non-significant trend. These results are consistent with the idea that while attachment patterns to mothers and fathers are relevant for symptom expression, avoidance to fathers may capture a specific dimension of relational stress associated with somatic symptomatology. This echoes findings that paternal relationships, particularly when characterized by emotional withdrawal, may be uniquely implicated in children’s self-regulatory difficulties and health-related complaints [28,58]. Longitudinal studies corroborate this evidence, showing that paternal psychological distress predicts a broad spectrum of child behavioral problems (e.g., hyperactivity, conduct, emotional, and peer-related difficulties), independently of maternal distress [59,60,61]. The third model, which included baseline RMSSD, BMI, and gender, explained a smaller proportion of variance in somatic symptoms, with gender being the only significant predictor—females reported higher somatic complaints. This finding is consistent with epidemiological data indicating higher somatic symptom reporting in girls and adolescent females [4], especially in the age group covered by this study, possibly reflecting both biological and psychosocial factors, including the differential expression of symptoms.

Our third hypothesis was partially supported. Although several studies suggest that lower vmHRV, often observed in the context of migraine and other chronic pain [62,63], is associated with impaired autonomic regulation and increased vulnerability to stress-related somatic symptoms [35], in our study, resting vmHRV was not directly associated with somatic symptoms. However, mediation analysis revealed a significant indirect pathway: lower RMSSD was associated with higher attachment avoidance toward fathers, which in turn predicted a greater severity of somatic symptoms. This finding suggests that, in this specific sample, reduced vagal tone may contribute to somatic symptom expression through its relationship with interpersonal functioning, specifically the quality of the father–child attachment, rather than through a direct physiological pathway. This interpretation is consistent with Porges’ polyvagal theory, which links vagally mediated HRV to social engagement and emotional regulation capacities [35]. Within pediatric migraine populations, insecure or avoidant attachment—particularly toward fathers—has been repeatedly associated with greater somatic symptom severity, increased headache frequency, and higher levels of psychological comorbidity [26,27,30]. The specificity of attachment avoidance to fathers as a mediator is noteworthy. Fathers are thought to play a distinctive role in fostering autonomy and emotion regulation in children [64] and in shaping children’s emotion regulation, pain processing, and psychosocial adjustment [26,27]. Furthermore, some studies have suggested that fathers’ perception of emotional unavailability may increase stress reactivity and compromise coping resources [26,27], potentially through a reduction in vagal tone. This is consistent with evidence that attachment insecurity could mediate the relationship between childhood adversity and migraine, indicating that relational stressors may exacerbate symptom perception [8,19,65,66]. Consistent with these findings, Williams (2017) reported that insecure attachment to fathers was linked to greater insecure attachment to mothers, suggesting a cumulative effect of parental relationships on anxiety symptoms in pediatric migraine and reinforcing the idea that insecurity in attachment to parents may amplify psychological vulnerability and symptom burden [30]. Therefore, considering the relational environment in the management and treatment of migraine in children could provide useful insights into the non-pharmacological management of this condition. From a clinical perspective, current consensus emphasizes cognitive behavioral therapy (CBT) and biofeedback as the gold-standard non-pharmacological interventions for pediatric migraine, both of which have shown efficacy in reducing headache frequency and severity [67,68]. However, few longitudinal studies have explored interventions that explicitly integrate caregiver–child relationships, despite evidence that attachment insecurity plays a key role in symptom maintenance and severity. As highlighted in previous works, future research should therefore examine multimodal approaches that combine established behavioral interventions with strategies targeting family dynamics and attachment, with the aim of optimizing outcomes in the management of pediatric migraine [13,30,65,67].

## 5. Conclusions

Taken together, our findings support a model in which autonomic regulation (indexed by resting HRV) may influence somatic symptom expression via interpersonal/relational mechanisms—specifically paternal avoidant attachment—rather than via a straightforward direct physiological effect. Beyond individual-focused approaches, the present findings highlight the importance of considering the family context when designing non-pharmacological interventions for pediatric migraine. Specifically, the observed associations between attachment patterns and somatic symptom severity suggest that the quality of the parent–child relationship may play a pivotal role in shaping symptom perception and regulation. Including an assessment of family dynamics and attachment-related processes could therefore strengthen interventions such as cognitive-behavioral therapy or biofeedback, which are currently the gold standard in the non-pharmacological management of pediatric migraine. This perspective is consistent with evidence that insecure attachment contributes to heightened vulnerability to stress and somatic complaints, and with research showing that relational functioning moderates the impact of physiological dysregulation on health outcomes. Thus, interventions that explicitly incorporate parental involvement or target the improvement of relational quality may offer added benefits by addressing not only individual coping mechanisms but also the socio-emotional environment in which children experience and manage migraine.

### Limitations and Implications

While the present study offers novel insights into the interplay between attachment, autonomic regulation and somatic symptoms in youth with migraine, several limitations should be acknowledged. First, the cross-sectional design prevents any conclusions regarding causal directionality. Although the mediation model revealed a significant indirect effect of HRV on somatic symptoms via attachment avoidance to fathers, the temporal sequence of these associations remains speculative. Longitudinal studies are necessary to determine whether lower vagal tone predisposes adolescents to adopt avoidant attachment patterns, or whether early relational experiences shape physiological regulation over time. Second, although the sample size was adequate for primary regression and mediation analyses, it limited the ability to formally test more complex interactions, such as moderated mediation. In addition, although age did not correlate with HRV indices, small associations emerged between age and avoidant attachment dimensions. We intentionally focused on a restricted age range (11–17 years) to minimize developmental and linguistic variability; however, this choice may have limited our ability to explore age-related differences in attachment or autonomic functioning. Future research should therefore recruit larger samples stratified by developmental stage to empirically examine the moderating role of age on the interplay between attachment patterns, autonomic regulation, and somatic symptoms, also considering the potential clinical implications of such differences. Third, HRV was assessed using a single short-term resting measure (RMSSD). While valid, this approach may not fully reflect the dynamic nature of autonomic regulation. Incorporating longer baseline recordings or reactivity paradigms could offer a more comprehensive view of vagal functioning with respect to relational patterns and somatic expressions. Thus, a future goal will be to examine the psychophysiological responses of these patients during an emotional activation task through projective identification with patterns of relating to caregivers as well as cognitive tasks. While this study provides preliminary support for an integrative model linking physiological regulation and attachment to somatic distress, future research should aim to replicate and expand these findings using longitudinal, developmentally sensitive, and methodologically diverse approaches. Finally, all participants were recruited from a single clinical service in Italy, which may limit the generalizability of the findings to other settings and populations.

## Figures and Tables

**Table 1:** Demographic and Psychophysiological Characteristics of the Sample.

Variable	N	%
*Gender*		
Male	24	39.3%
Female	37	60.7%
	**M**	**SD**
Age (years)	13.9	1.98
CSI-24	24.8	13.5
ECR-RC Anxiety—Mother	8.28	3.34
ECR-RC Avoidance—Mother	15.2	6.02
ECR-RC Anxiety—Father	8.19	3.55
ECR-RC Avoidance—Father	17.1	5.97
RMSSD—Baseline (ms)	40.3	21.2
Body Mass Index (BMI)	23.7	5.35

Note. Values are presented as means (M) and standard deviations (SD). CSI-24 = Children Somatization Inventory-24; ECR-RC = Experiences in Close Relationships–Revised Child Version; RMSSD = Root Mean Square of Successive Differences.

**Table 2:** Associations between somatic symptoms, attachment patterns to mother and father, and resting vmHRV.

	CSI-24	ECR-RC Anxiety Mother	ECR-RC Avoidance Mother	ECR-RC Anxiety Father	ECR-RC Avoidance Father	RMSSD—BASELINE
CSI-24	—					
ECR-RC Anxiety Mother	0.422 ***	—				
ECR-RC Avoidance Mother	0.465 ***	0.450 ***	—			
ECR-RC Anxiety Father	0.334 *	0.438 ***	0.153	—		
ECR-RC Avoidance Father	0.444 **	0.271 *	0.651 ***	0.406 **	—	
RMSSD—Baseline	0.034	−0.170	−0.197	−0.170	−0.330 *	—

Note. * *p* < 0.05, ** *p* < 0.01, *** *p* < 0.001.

**Table 3:** Linear Regression: attachment to mother and gender as Predictors of CSI-24.

Model Adjustment Measures				
**Model**	**R**		**R^2^**	
1	0.536		0.288	
**Predictor**	**Estimate**	**SE**	**t**	***p***
Intercept	6.787	5.291	1.28	0.205
ECR-RC Anxiety Mother	1.066	0.506	2.10	0.040
ECR-RC Avoidance Mother	0.694	0.290	2.39	0.020
GENDER:				
M—F	−3.530	3.202	−1.10	0.275

Model Coefficients—CSI-24. Note. Models estimated using sample size of N = 61.

**Table 4:** Linear Regression: Attachment to Father and gender as Predictors of CSI-24.

Model Adjustment Measures				
**Model**	**R**		**R^2^**	
1	0.499		0.249	
**Predictor**	**Estimate**	**SE**	**t**	***p***
Intercept	7.965	6.016	1.32	0.191
ECR-RC Anxiety Father	0.863	0.515	1.68	0.099
ECR-RC Avoidance Father	0.669	0.327	2.05	0.046
GENDER:				
M—F	−4.574	3.586	−1.28	0.208

Model Coefficients—CSI-24. Note. Models estimated using a sample size of N = 57.

**Table 5:** Linear Regression: vmHRV, BMI, and Gender as Predictors of CSI-24.

Model Adjustment Measures				
**Model**	**R**		**R^2^**	
1	0.287		0.0825	
**Predictor**	**Estimate**	**SE**	**t**	***p***
Intercept	25.0559	9.5964	2.611	0.012
RMSSD—Baseline	−0.0408	0.0934	−0.437	0.664
BMI	0.1626	0.3290	0.494	0.623
GENDER:				
M—F	−7.2141	3.5815	−2.014	0.049

Model Coefficients—CSI-24. Note. Models estimated using sample size of N = 55.

**Table 6:** Attachment patterns to mothers and fathers as potential mediators of the effect of resting vmHRV on somatic symptoms.

Models Info									
Mediators Models			m1		ECR-RC Avoidance Father ~ RMSSD—BASELINE				
Full Model			m2		CSI-24 ~ ECR-RC Avoidance Father + RMSSD—BASELINE				
Indirect Effects			IE 1		RMSSD—BASELINE ⇒ ECR-RC Avoidance Father ⇒ CSI-24				
Sample size			N		52				
**Indirect and Total Effects 95% C.I.**									
**Type**	**Effect**	**Estimate**		**SE**	**Lower**	**Upper**	**β**	**z**	***p***
Indirect	RMSSD—BASELINE ⇒ ECR-RC Avoidance Father ⇒ CSI-24	−0.1050		0.0496	−0.2159	−0.0355	−0.1661	−2.116	0.034
Component	RMSSD—BASELINE ⇒ ECR-RC Avoidance Father	−0.0946		0.0376	−0.1568	−0.0367	−0.3298	−2.519	0.012
	ECR-RC Avoidance Father ⇒ CSI-24	1.1100		0.2844	0.5955	1.6710	0.5037	3.902	<0.001
Direct	RMSSD—BASELINE ⇒ CSI-24	0.1302		0.0816	−0.0717	0.2997	0.2060	1.596	0.111
Total	RMSSD—BASELINE ⇒ CSI-24	0.0217		0.0851	−0.1784	0.1835	0.0343	0.254	0.799

Note. Confidence intervals computed with the bias-corrected bootstrap method. Note. Betas are completely standardized effect sizes.

## Data Availability

The data supporting the findings of this study are available from the corresponding author upon reasonable request, provided that a valid scientific rationale is given.

## Abbreviations

The following abbreviations are used in this manuscript:

ACEs	Adverse Childhood Experiences
ANS	Autonomic Nervous System
BMI	Body Mass Index
CBT	Cognitive Behavioral Therapy
CSI-24	Children’s Somatization Inventory—24
ECR-RC	Experiences in Close Relationships—Revised Child Version
ECG	Electrocardiogram
GI	Gastrointestinal
HRV	Heart Rate Variability
ICHD-3	International Classification of Headache Disorders, 3rd edition
RMSSD	Root Mean Square of Successive Differences
vmHRV	Vagally Mediated Heart Rate Variability
